# Novel gene expression patterns along the proximo-distal axis of the mouse embryo before gastrulation

**DOI:** 10.1186/1471-213X-7-8

**Published:** 2007-02-15

**Authors:** Stephen Frankenberg, Lee Smith, Andy Greenfield, Magdalena Zernicka-Goetz

**Affiliations:** 1 The Wellcome Trust/Cancer Research UK Gurdon Institute of Cancer and Developmental Biology, Tennis Court Rd, Cambridge CB2 1QR, UK; 2 Inserm, U384, Clermont-Ferrand, F-63001, France; 3 MRC Mammalian Genetics Unit, Harwell, Didcot OX11 0RD, UK

## Abstract

**Background:**

To date, the earliest stage at which the orientation of the anterior-posterior axis in the mouse embryo is distinguishable by asymmetric gene expression is shortly after E5.5. At E5.5, prospective anterior markers are expressed at the distal tip of the embryo, whereas prospective posterior markers are expressed more proximally, close to the boundary with the extraembryonic region.

**Results:**

To contribute to elucidating the mechanisms underlying the events involved in early patterning of the mouse embryo, we have carried out a microarray screen to identify novel genes that are differentially expressed between the distal and proximal parts of the E5.5 embryo. Secondary screening of resulting candidates by *in situ *hybridisation at E5.5 and E6.5 revealed novel expression patterns for known and previously uncharacterised genes, including *Peg10*, *Ctsz1*, *Cubilin*, *Jarid1b*, *Ndrg1*, *Sfmbt2*, *Gjb5*, *Talia *and *Plet1*. The previously undescribed gene *Talia *and recently identified *Plet1 *are expressed specifically in the distal-most part of the extraembryonic ectoderm, adjacent to the epiblast, and are therefore potential candidates for regulating early patterning events. *Talia *and the previously described gene *XE7 *define a gene family highly conserved among metazoans and with a predicted protein structure suggestive of a post-transcriptional regulative function, whilst *Plet1 *appears to be mammal-specific and of unknown function.

**Conclusion:**

Our approach has allowed us to compare expression between dissected parts of the egg cylinder and has identified multiple genes with novel expression patterns at this developmental stage. These genes are potential candidates for regulating tissue interactions following implantation.

## Background

At 5.5 days of development (E5.5) the mouse egg cylinder appears radially symmetrical about its proximo-distal axis with respect to known molecular markers and to the arrangement of its three principle tissue layers – epiblast, extra-embryonic ectoderm and visceral endoderm. However, shortly after E5.5 the first molecular asymmetries that determine the anterior-posterior axis begin to emerge. These involve movement of a subset of visceral endoderm cells, anterior visceral endoderm (AVE), located at the distal tip of the egg cylinder towards the future anterior side [1-5]. Subsequent to this, molecular markers with a previously radial distribution near the embryonic-extra-embryonic boundary become restricted to the future posterior side at the site of the emerging primitive streak [6]. In this way the proximo-distal signaling anticipates the anterior-posterior patterning [6,7]. Patterning thus occurs through a combination of tissue interactions and cell movements [reviewed [8]].

The stages of mouse development between implantation and the gastrulating egg cylinder have been relatively little studied. This is due partly to the relative inaccessibility of embryos within the uterine deciduae during this time, and partly to their relatively poor development in culture compared with preimplantation and gastrula stages. More recently, much attention has been focused on the events preceding gastrulation and their relation to earlier preimplantation development, providing an incentive to identify novel genes with restricted expression patterns during these stages.

Several recent microarray screens have focused on stage-specific expression in pre-implantation embryos [9-11], whilst other screening strategies have targeted specific tissues of post-implantation embryos [12-15]. In an effort to identify new genes that are differentially expressed along the proximo-distal axis and may have roles in early pre-gastrula patterning events, we employed microarray analysis to compare gene expression between proximal and distal halves of the E5.5 egg cylinder. The proximal half includes extraembryonic ectoderm and the proximal portion of the visceral endoderm, while, the distal half includes the epiblast and the distal portion of the visceral endoderm. After secondary screening by *in situ *hybridisation, we identified both known and novel genes with previously unreported differential expression in the early mouse egg cylinder.

## Results

**Figure 1:**
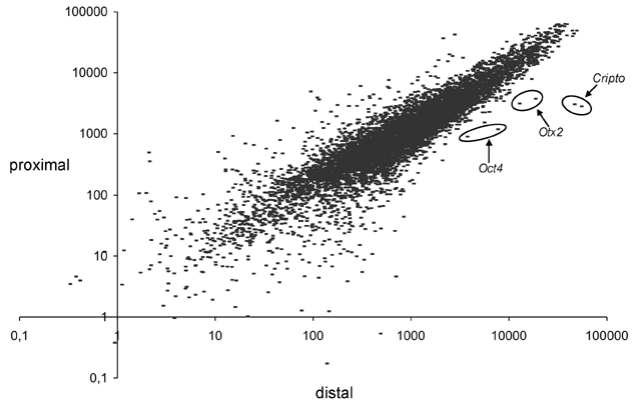
Scatter plot of microarray data comparing relative levels of gene expression for proximal and distal parts of the E5.5 egg cylinder (x – distal, y – proximal). Points representing several previously published genes (*Oct4*, *Otx2*, *Cripto*) that display clear differential expression between proximal and distal parts are circled.

**Figure 2:**
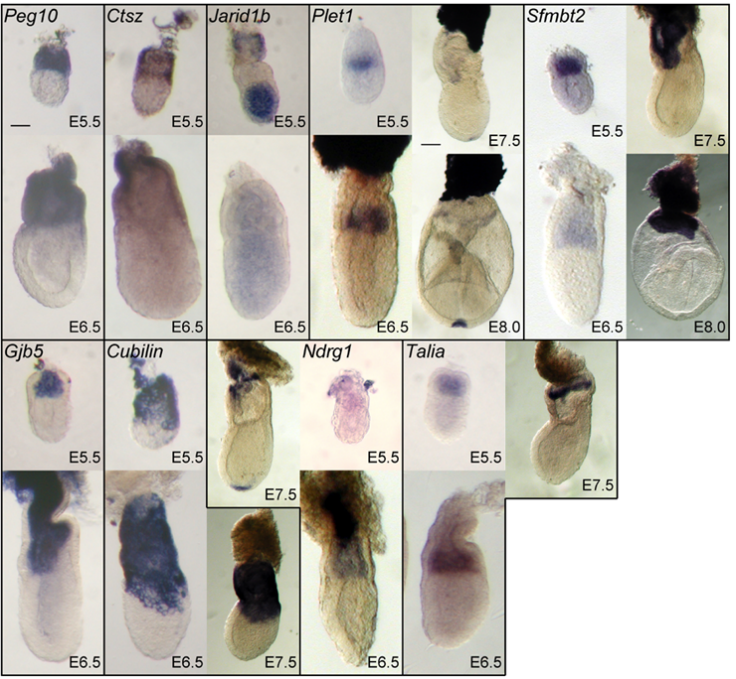
Whole-mount *in situ *hybridisation of genes identified in microarray screen with differential expression patterns at stages as indicated. The two scale bars represent respectively 100 μm for all E5.5 and E6.5 images and 200 μm for all E7.5 and E8.0 images.

**Figure 3:**
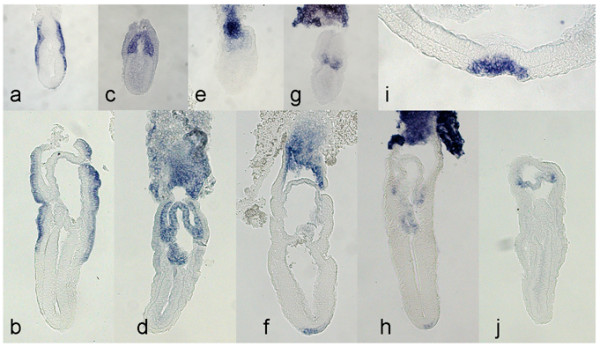
Longitudinal sections of embryos after whole-mount *in situ *hybridisation, showing expression of: *Cubilin *at E6.5 (a) and E7.5 (b); *Sfmbt2 *at E6.5 (c) and E7.5 (d) (note that due to the distorted shape of the specimen this section does not fully pass through the lumen of the proamniotic cavity and the distal-most group of *Sfmbt2*-expressing cells in fact forms part of the extraembryonic ectoderm near the anterior amniotic fold); *Ndrg1 *at E6.5 (e) and E7.5 (f); *Plet1 *at E6.5 (g), E7.5 (h) (note also that the distal-most extraembryonic expression represents extraembryonic ectoderm near the anterior amniotic fold) and in the node at E8.0 (i); and *Talia *at E7.5 (j).

We compared gene expression between the proximal and distal halves of the E5.5 egg cylinder by microarray analysis to identify genes with previously unreported differential expression at this stage of development. A scatter plot of expression levels in proximal and distal segments reveals a large number of genes that putatively show such differential expression (Fig. 1). Several genes with previously reported differential expression in the egg cylinder showed relative hybridisation levels consistent with such expression patterns. These included *Otx2 *[16], *Cripto *[17], *Dnmt3b *[18] and *Oct4 *[19] distally, and *Gjb3 *[20], *Pem *[21], *Igf2 *[22] and *H19 *[22] proximally. We therefore wished to test whether other previously uncharacterised genes were also differentially expressed. We selected 40 genes, partly on the basis of differential and absolute levels of hybridisation in the microarray and partly on their likely involvement in developmental pathways, and further screened these genes by *in situ *hybridisation. Of these, we successfully identified 9 genes with previously unreported differential expression at E5.5 and E6.5, while the remainder showed either undetectable or ubiquitous expression. The 9 genes and their expression patterns are shown in Figures 2 and 3 and summarised in Table 1. *In situ *hybridisation was extended to later stages from E7.5 to E9.5 for several genes, including *Cubilin, Jarid1b, Sfmbt2, Ndrg1, Talia *and *Plet1*. However no tissue specific-expression within embryonic tissues was identified for any of these gene later than E8.5 (not shown), aside from continued extraembryonic expression for *Cubilin*, *Sfmbt2, Ndrg1*, and *Plet1*.

**Table 1:** Summary of genes with restricted expression patterns

NIA clone ID	Gene name	Tissue with specific expression
H3001E07-3	*Peg10*	proximal VE
H3017E04-3	*Ctsz*	proximal VE
H3004E08-3	*Cubilin*	proximal VE
H3041C04-3	*Jarid1b*	epiblast + ectoplacental cone
H3031C12-3	*Ndrg1*	extraembryonic ectoderm + node
H3001A06-3	*Sfmbt2*	extraembryonic ectoderm
H3016G11-3	*Gjb5*	extraembryonic ectoderm
H3001D07-3	*Talia*	extraembryonic ectoderm
H3011D11-3	*Plet1*	extraembryonic ectoderm + node

Expression patterns fell into several broad categories. *Peg10*, *Ctsz *and *Cubilin *were expressed in the visceral endoderm mainly within the proximal or "extraembryonic" part of the egg cylinder. The expression of *Cubilin *also extended into the distal portion of the egg cylinder in the form of two lateral "wings" overlying the epiblast. Tissue sectioning showed that this expression corresponds to the distal extent of cuboidal visceral endoderm cells (Fig. 3a, b).

**Figure 4:**
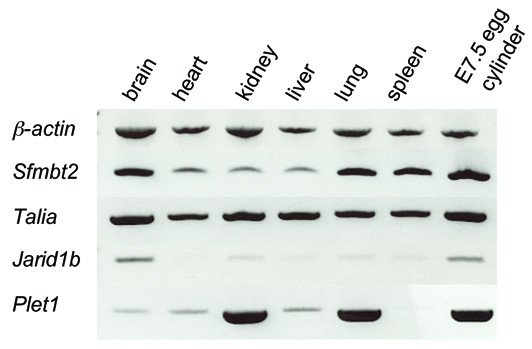
RT-PCR of selected genes showing expression in adult tissues compared with whole E7.5 embryo (negative image). *β-actin *expression (first row) was used as a control.

*Jarid1b *(also called *Plu-1 *or *Rb-Bp2*) was expressed strongly in the epiblast at E5.5 but more weakly and ubiquitously at E6.5 (Fig. 2) and later stages (not shown). RT-PCR suggested a higher level of expression in whole E7.5 embryos than in adult tissues, with the exception of strong expression in the brain (Fig. 4).

Two genes, and *Gjb5 *and *Sfmbt2*, were expressed throughout the extraembryonic ectoderm. In sectioned embryos, *Sfmbt2 *expression appeared uniform within the chorionic ectoderm and also within the ectoplacental cone until at least E7.5 (Fig. 3c, d). *Sfmbt2 *expression was also detected in all adult tissues tested but was substantially stronger in brain, lung and spleen compared with heart, kidney and liver (Fig. 4).

*Ndrg1 *was expressed uniformly throughout the extraembryonic ectoderm but more strongly within the labyrinth of the ectoplacental cone in all stages examined (Fig. 2, Fig. 3). At E7.5 and E8.0 (Fig. 2, 3f), expression was also present in the node, while becoming weaker within the chorionic ectoderm. No specific expression in other embryonic tissues was detected later at either E8.5 or E9.5 (not shown).

*Plet1 *was also specifically expressed in the extraembryonic ectoderm, but restricted to its distal-most part as early as E5.5 as well as a separate domain of much stronger expression within the ectoplacental cone. The distally-restricted expression persisted, but becoming weaker and restricted to the peripheral chorion, until at least E8.5 (Fig. 2, 3g–h). Expression was also detected within the ventral layer of the node (Fig. 3i).

### Talia

A previously undescribed gene corresponding to clone H3001D07-3 was also uniformly expressed in the extraembryonic ectoderm at E5.5, and by E6.5 was also restricted to its more distal part, adjacent to the epiblast. Although levels appeared lower at later stages, expression appeared to be strongest around the perimeter of the distal part of the chorionic ectoderm at E7.5 (Fig. 3j). Expression was detected ubiquitously in all adult tissues examined by RT-PCR (Fig. 4).

A BLAST search of genomic databases identified the gene as mapping to region XA2 of the murine X-chromosome. A human orthologue was also identified in the syntenic region Xq24 of the human X-chromosome and in the marsupial *Monodelphis domestica *by sequence database searches, indicating that the gene is conserved in mammals. The 5'-most part of the predicted transcript also showed homology to another previously described human gene, *XE7*, which maps to Xp22.3 of the X-chromosome and was originally identified as a pseudoautosomal gene that escapes X inactivation [23,24] and encodes a cell surface glycoprotein expressed in trophoblast and lymphocytes [25]. The murine gene represented by clone H3001D07-3 we thus named *Talia *(a Polish word for "waistline") to reflect its belt-like expression pattern in the distal part of the extraembryonic ectoderm.

### Sequence analysis of *Talia *and *XE7*

**Figure 5:**
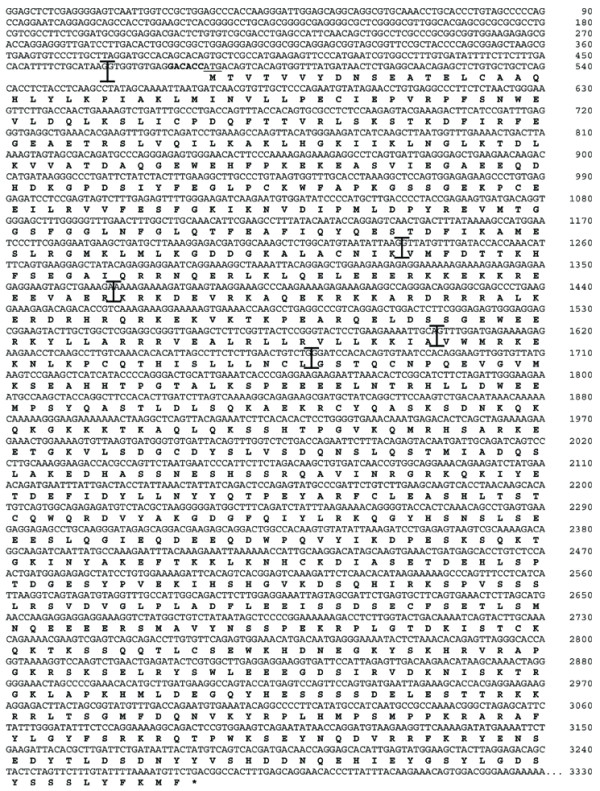
Predicted coding region of mouse *Talia*. The start ATG codon is underlined, immediately following a Kozak translation initiation motif (bold). Exon-exon boundaries are indicated by I-shaped separators.

**Figure 6:**
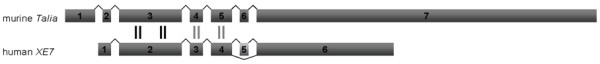
Comparison of the exon structure and homology of mouse *Talia *with human *XE7*. Homology between exons is indicated by dashed vertical bars, with the highest sequence conservation between exon 3 of *Talia *and exon 2 of *XE7 *(darker bars). Two splice variants have been demonstrated for *XE7*, as indicated. The inclusion of exon 5, which contains two in-frame stop codons, is predicted to result in a truncated protein [23]. (Relative scaling of exon sizes are only approximate.)

Analysis of genomic sequence data and ESTs revealed 7 exons for murine *Talia *with a predicted mRNA length of 6152 nucleotides (Fig. 5, 6). Promoter prediction software [26] indicated an additional promoter and transcription initiation site within the 5' part of exon 7, suggesting alternative primary transcripts. However probes specific for sequences either 5' or 3' of this position showed indistinguishable expression patterns by *in situ *hybridisation (not shown), suggesting that either the latter putative promoter is non-functional or that both are functional but share common regulatory mechanisms.

**Figure 7:**
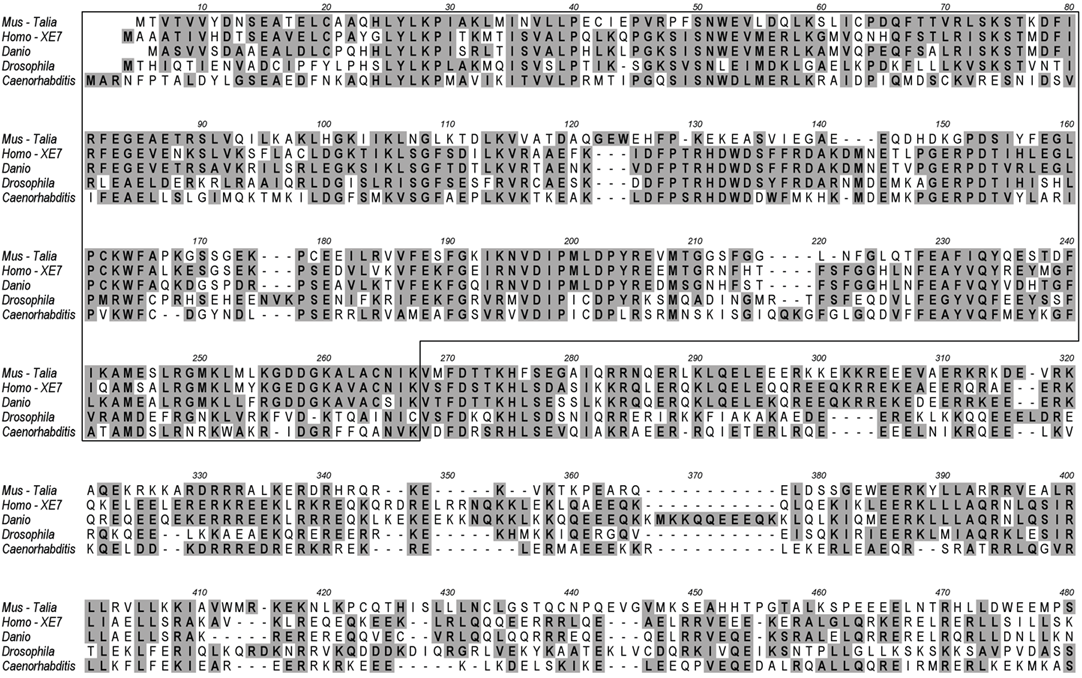
(a) Alignment of predicted amino acid sequences from *Talia*, *XE7 *and homologues from other metazoans within the region of homology. The most highly conserved region, corresponding to exon 3 of *Talia*, is boxed. Highly conserved amino acids (in > 50% of sequences) are highlighted. Human *TALIA *(not shown) includes two "stop" codons at the equivalent of positions 234 and 248 in the alignment. Accession numbers of nucleotide sequences, from which protein sequences were predicted, were: *Talia *(*Mus musculus*) – AW261571; *XE7 *(*Homo sapiens*) – NM_005088; *Danio rerio *– NM_200682; *Drosophila melanogaster *– NM_169093; *Caenorhabditis elegans *– NM_066250.

*Talia *and *XE7 *showed homology between exons 3–5 of murine *Talia *and exons 2–4 of human *XE7 *(Fig. 6, 7). BLAST searches revealed numerous ESTs derived from various tissue sources that were concluded to represent murine *Xe7*, despite its apparent absence from current genomic databases. BLAST searches also identified ESTs representing genes with homology to this conserved region at the amino acid level from each of a broad range of metazoans, including *Gallus gallus*, *Xenopus spp*., *Danio rerio*, *Drosophila melanogaster*, *Caenorhabditis elegans *and *Hydra magnipapillata*, suggesting a highly conserved function for this gene among metazoans. Alignment of sequences indicated amino acid conservation was maximal within the region corresponding to exon 3 of *Talia *(Fig. 6).

*Talia*/*TALIA *appears to be specific to mammals, being more divergent than *XE7 *from homologues in other metazoans, and apparently represents the only conserved evolutionary duplication of the ancestral gene detectable in genomic databases. Functional orthologues of *Talia *in pig and rat are supported by EST evidence, however human *TALIA *is apparently not expressed, as no corresponding ESTs were identified in existing databases. Furthermore, the human genomic sequence (LOC139516) contains two premature in-frame stop codons (corresponding to positions 234 and 348 in Fig. 6), suggesting that it is non-functional. Conversely, many more ESTs for human *XE7 *appeared to be present in databases compared with those for murine *Xe7*, raising the possibility that mammalian *XE7 *and *TALIA *have overlapping roles and may variously substitute for one another in different species.

**Figure 8:**
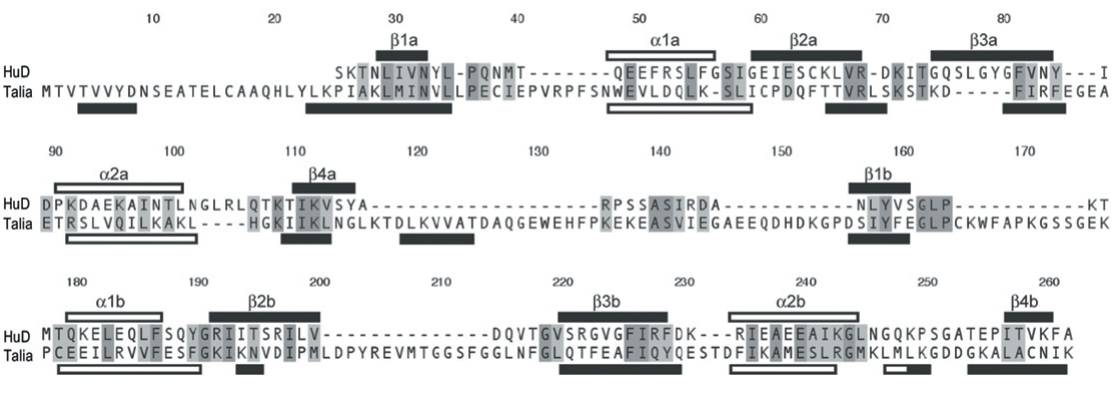
Predicted primary and secondary structures of Talia aligned with the known structure of HuD protein [28]. Dashes are introduced for the purpose of alignment. Talia shows a similar arrangement of predicted α-helices (open rods) and β-strands (closed rods) to that of HuD, which contains two repeated β1-α1-β2-β3-α2-β4 motifs separated by a linker region, and strongly suggests a similar function to HuD in binding to the 3' UTRs of mRNAs. High divergence in primary structure, however, suggests they differ in their target sequences.

Voland *et al*. [25] identified several potential functional motifs within XE7, including a putative leucine zipper domain, transmembrane domain and N-glycolsylation sites. However these motifs are not conserved in either *Talia *or *XE7 *from different species. Moreover, comparison of the predicted amino acid sequences of *Talia *and *XE7 *with protein databases revealed a significant similarity within the conserved region to the first two tandem RNA recognition motifs (RRMs) of Hu proteins, which bind to adenosine-uridine-rich elements (AREs) in the 3' untranslated regions of mRNAs [27]. The predicted secondary structure of this region of Talia is similar to the known secondary structure of HuD (Fig. 8), which consists of a β1-α1-β2-β3-α2-β4 topology within each RRM [28]. However, the residues of HuD that were shown to interact with ARE sequences are not conserved in Talia or XE7, suggesting that they interact with different target sequences.

## Discussion

This study identified a number of genes with previously unreported differential expression in the early postimplantation mouse embryo. Several represent new candidates for genes involved in tissue interactions controlling early events upon implantation. Three genes identified in the screen – *Peg10, Ctsz *and *Cubilin *– showed specific expression in the visceral endoderm of the egg cylinder. The human orthologue of *Peg10 *was originally identified as a paternally expressed imprinted gene with homologies in two open reading frames to gag and pol proteins of some vertebrate retrotransposons [29]. It forms part of a novel imprinted gene cluster on human chromosome 7 [30] and mouse chromosome 6 [31] and has been shown to have oncogenic activity in hepatoma cells, suggesting a role in cell proliferation [32]. Cathepsin Z, encoded by *Ctsz*, is a member of the C1 family of cysteine proteases of unknown function. The gene lies proximal to a cluster of imprinted genes but is not itself imprinted [33]. Previously, *Ctsz *was reported as ubiquitously expressed in adult tissues [34]. This study, however, shows that *Ctsz *has tissue-specific expression at least in the early post-implantation embryo.

*Cubilin *encodes a multiligand endocytic receptor involved in uptake of low density lipoproteins and is present in absorptive epithelia of the ileum, kidney and visceral yolk sac [reviewed in: [35,36]]. Its lateral endodermal expression overlying the epiblast at both E6.5 and E7.5 is of interest as it may reflect the movements of this tissue during early gastrulation (Thomas et al, 1998; Perea-Gomez et al, 2001). While no antero-posterior asymmetry was evident in the expression of *Cubilin *alone, it would be interesting to investigate the degree to which it overlaps with AVE markers such as *Lefty1 *[37,38], *Cer1 *[39] and particularly *Dkk1*, which is expressed in the more proximal part of the embryonic VE from E5.25 and of the later AVE [40].

*Jarid1b *showed expression in epiblast and the outer part of the ectoplacental cone at E5.5, however by E6.5 epiblast expression was weak or undetectable. *Jarid1b *encodes a nuclear protein that was originally identified in human breast cancer cell lines [41]. In normal adult tissues of human and mouse, expression is largely restricted to testis and ovary [41,42]. In E12.5-15.5 mouse embryos, expression occurs in a spatially restricted pattern that overlaps with those of Bf-1 and Pax-9 [42,43], with which PLU-1 has been shown to interact [44].

Three genes – *Ndrg1*, *Sfmbt2 *and *Gjb5 *– identified in the screen showed specific expression throughout the extraembryonic ectoderm at both E5.5 and E6.5. *Ndrg1 *was originally identified by its upregulation in *N-myc *deficient mice [45]. While its precise function remains obscure, it has been reported to be involved in cell growth and differentiation [45-48]. Sfmbt2 is related to the *Drosophila *polycomb group of transcriptional repressors, which regulate homeotic and other genes [49-51]. The closely related murine gene *Sfmbt1 *(= *Sfmbt*) was shown to be highly expressed in adult testis, with a much lower expression in other adult and late embryonic tissues [52]. *Sfmbt2 *has not been characterised, however database searches of matching ESTs indicate expression in testis and germ cells, suggesting that Sfmbt1 and Sfmbt2 may have related roles.*Gjb5*, which showed very strong specific expression in the extra-embryonic ectoderm, encodes one of a large family of gap junction proteins. A number of other gap junction proteins also show spatially restricted expression during early post-implantation development, indicative of a role in establishing communication compartments [53,54], whilst *Gjb5 *expression has previously been shown in preimplantation embryos [55]. While the roles of gap junctions during early development remain unclear, it is possible they may help to facilitate the transduction of signalling molecules within tissues.

Two genes – *Plet1 *and *Talia *– showed localised expression in the extraembryonic ectoderm that may suggest a role in the interactions between epiblast and extraembryonic ectoderm. Signals from this region of the egg cylinder are believed to regulate proximo-distal patterning of the epiblast via such factors as Nodal, Cripto and Otx2 [5,17,56-62]. Proximo-distal signaling contributes to anterior-posterior patterning via asymmetric cell movements that position the AVE opposite the site of primitive streak formation. The specific identity of extraembryonic ectoderm adjacent to the epiblast is thought to be regulated by Fgf4 signaling via activation of the Erk pathway (reviewed [63]). Thus it is likely that expression of *Plet1 *and *Talia *is regulated downstream of this signaling, and may have roles in specifying this identity. In particular, the restricted expression of *Plet1 *from E5.5 appears to be earlier than has been reported for other genes such as *Eomes *[64] and *Bmp4 *[65], suggesting that *Plet1 *expression may be directly regulated by this pathway. Recently identified by its trophoblast-specific expression at later stages of mouse, pig and human [66,67], no functional motifs are evident to suggest a role for the protein.

By contrast, we have shown from its predicted tertiary structure that Talia is likely to function as a post-transcriptional regulator due to its similarities with HuD, an RNA-binding protein shown to have a role in specifying neural cell identity [68]. Interestingly the role of Talia is possibly substituted by a homologue, XE7, in humans. The high conservation of orthologues of *Talia*/*XE7 *amongst metazoans further supports an essential role in development.

## Conclusion

This study demonstrated for the first time the application of a microarray strategy for identifying genes that are differentially expressed between dissected parts of the early post-implantation mouse embryo. It successfully identified several genes, both known and previously uncharacterised, with novel expression patterns in the early mouse post-implantation embryo. Some of these, such as *Talia *and *Plet1*, will be of particular interest for further analysis, particularly with respect to possible roles in specifying the identity of extraembryonic ectoderm adjacent to the epiblast or in signaling to the proximal epiblast.

## Methods

### Tissue collection

Fifty E5.5 embryos from F1 (C57/BL6 × CBA) females crossed with F1 males were collected into M2 medium. After removal of Reichert's membrane, embryos were cut at the embryonic-extraembryonic boundary into proximal and distal halves, using a finely drawn glass needle, respectively pooled, frozen on dry ice and stored at -80C until RNA extraction.

### Preparation of labelled target cDNA

Total RNA was extracted using an RNeasy Mini Kit (Qiagen) according to the manufacturer's instructions. Target cDNA synthesis and single primer amplification (SPA) followed by labelling with Cy5- or Cy3-dCTP were performed as previously described [69]. Labelled target cDNA was hybridised to an array of plates 3001–3048 of the NIA 15 K mouse cDNA set [70] spotted in duplicate (4608 ESTs, 9216 spots) on CMT-GAPS-coated slides (Corning) and analysed as previously described [69]. Candidate genes were selected on the basis of both ratio and absolute difference in hybridisation level of each of the target cDNAs. Clone names used below can be identified via the NIA/NIH Mouse Genomics website [71].

### Whole mount *in situ *hybridisation

E5.5 and E6.5 embryos were collected as above and, after removal of Reichert's membrane, fixed in 4% paraformaldehyde in phosphate buffered saline overnight at 4°C. DNA templates were prepared by PCR from plasmid DNA using T3, SP6 and T7 promoter-specific primers. Plasmid templates for each probe were either transcribed directly from NIA clones (corresponding to those used in the array) or were cloned by RT-PCR from E6.5 embryo mRNA into the *Eco*RI and *Sal*I sites of pBluescript II KS(+). Respective forward and reverse primers for the latter were: *Jarid1b*, 5'-GGAATTCGGGTTGCTTGCTTCTGCTTCTTC and 5'-GCGTCGACATCAGGGGAAACTGGTATCGGC; *Gjb5*, 5'-GGAATTCCTACCTCTTCCACGCATTCTATCC and 5'-GCGTCGACAGGCATTTGCTCATCGGTGC; *Sfmbt2*, 5'-GGAATTCGTCTCTGGGGACATCTACTGCTTG and 5'-GCGTCGACTGCTCTGCCTCGGTTCTGTG; *Ndrg1*, 5'-GGAATTCGAGAGAGAGAGGCAGGAAAGTTGG and 5'-GCGTCGACTACAAACCCAGTCAGCAGGAGG; *Cubilin*, 5'-GGAATTCAACCTTGCCCGTGTTCTATTCC and 5'-GCGTCGACTGAAGACCCGATTTGATGAAGC; *Talia *(exon 7), 5'-GGAATTCATCCTGGCACATCAATAATGGC and 5'-GCGTCGACAAGTAACCCCACAGACTGACATCC; *Talia *(exons 3–4), CATTTTCTGCATAAGGTGGTGTGAGGAC and GCCTGATAGCATCGCTTCTCTGCC;*Plet1*, 5'-GGAATTCCTGAAAGCAGTGAAGGAGGACG and 5'-GCGTCGACCACGCAGGATGGATGGACTAAG.

Digoxygenin-labelled antisense RNA probes were prepared using an Ambion MegaScript transcription kit (SP6, T3 or T7) according to the manufacturer's instructions. *In situ *hybridisation was performed as described by Wilkinson and Nieto [72].

### Sequence analysis

Genomic structure was analysed using the Genomatix web-based sequence analysis software[26]. ESTs were searched using BLAST and sequence alignments performed using MacVector. Protein secondary structure prediction and similarity searches were performed using the 3D-PSSM web-based software [73,74].

### Analysis of expression by RT-PCR

Total RNA was extracted from adult female mouse tissues and pooled E7.5 egg cylinders using the RNeasy Mini Kit (QIAGEN) according to the manufacturers instructions. Oligo-dT(20)-primed first strand cDNA was prepared from 0.5 μg of total RNA in a 10-μl reaction volume using SuperScript III (Invitrogen) at 50°C for 1 hour according to the manufacturer's instructions. 0.5 μL of template was then used for each 15-μL PCR reaction mixture containing 0.05 Units/μL GoTaq polymerase (Promega), 1× supplied PCR buffer, 0.25 mM dNTPs and 0.5 μM each of forward and reverse gene-specific primers. 35 cycles of PCR were performed comprising 15 seconds denaturation (94°C), 15 seconds annealing (55°C) and 30 seconds extension (72°C). 8 μL of each PCR product was separated by electrophoresis in a 1.5% agarose gel containing ethidium bromide.

## Authors' contributions

SF performed all collection and experimental work on mouse embryos and writing of the manuscript. SF, with the crucial assistance of LS, performed the microarray screen and data analysis. AG was involved in facilitating and coordinating the microarray experiments. MZG was involved in the conception and coordination of this project. All authors approved the final manuscript.
